# Identification of knee osteoarthritis disability phenotypes regarding activity limitation: a cluster analysis

**DOI:** 10.1186/s12891-020-03260-y

**Published:** 2020-04-13

**Authors:** Mantana Vongsirinavarat, Patcharin Nilmart, Sirikarn Somprasong, Benjawan Apinonkul

**Affiliations:** 1 Faculty of Physical Therapy, Mahidol University, Putthamonthon, Nakhon Pathom, 73170 Thailand; 2 Department of Physical Therapy, School of Allied Health Science, Walailak University, Nakhon Si Thammarat, Thailand

**Keywords:** Activities of daily living, Chronic limitation of activity, ICF, Knee, Osteoarthritis, Cluster analysis

## Abstract

**Background:**

Studies have reported the subtypes of individuals with knee osteoarthritis (OA) attempting to cluster this heterogonous condition. Activity limitations are commonly used to set goals in knee OA management and better identify subgroups based on level of disability in this patient population. Therefore, the objective of this study was to identify those activity limitations which could classify the disability phenotypes of knee OA. The phenotypes were also validated by comparing impairments and participation restrictions.

**Methods:**

Participants comprised individuals with symptomatic knee OA. They were interviewed and undertook physical examination according to a standard evaluation forms based on the International Classification of Functioning, Disability and Health (ICF) model. Cluster analysis was used to determine those activity limitations which could best classify the phenotypes of knee OA. To validate the clustered variables, comparisons and regression analysis were performed for the impairments consisting of pain intensity, passive range of motion and muscle strength, and the participation restrictions included the difficulty level of acquiring goods and services and community life.

**Results:**

In all, 250 participants with symptomatic knee OA were enrolled in the study. Three activity limitations identified from data distribution and literature were used as the cluster variables, included the difficulty level of maintaining a standing position, timed stair climbing and 40-m self-paced walk test. The analysis showed four phenotypes of individuals with knee OA according to the levels of disability from no to severe level of disability. All parameters of impairment and participation restrictions significantly differed among phenotypes. Subgroups with greater disability experienced worse pain intensity, limited range of motion (ROM), muscle power and participation restriction levels. The variance accounted for of the subgroups were also greater than overall participants.

**Conclusion:**

The results of this study emphasized the heterogeneous natures of knee OA. Three activity limitations identified could classify the individuals with symptomatic knee OA to homogeneous subgroups from no to severe level of disability. The management plan, based on these homogeneous subgroups of knee OA, could be designated by considering the levels of impairments and participation restrictions.

## Background

Knee osteoarthritis (OA) is a condition with great heterogeneity by its nature. Despite similar structural involvements among people with knee OA, different outcomes of management were observed [1–3]. Classifying this population in homogeneous subgroups might lead to more direct and specific treatments [4]. Related studies have attempted to identify the common phenotypes of knee OA using various methods such as cluster analysis [5], latent class analysis [6], and predefined [7] methods. Considering the definition of phenotype as “the observable properties of an organism that are produced by the interaction of the genotype and the environment” [8], studies had attempted to identify the phenotypes of individuals with knee OA. Impairment physiognomies commonly used as phenotypic variables in research studies involve clinical findings such as pain sensitivity [9, 10], knee alignment [7], and gait parameters [11].

Considering the International Classification of Functioning, Disability and Health (ICF) framework, not only impairment but activity limitation and participation restriction were also affected by the pathology of knee OA [12, 13]. However, participation restriction is usually individualized and depends largely on personal as well as environmental factors. Therefore, functional disability or activity limitation is more commonly used to guide goal setting in knee OA management in rehabilitation. Related studies regarding physical therapy interventions usually evaluated the physical functions of these patients [14–17].

Using activity limitation to identify the phenotypes among patients with knee OA would lead to more specific levels of disability related to the physical therapy treatment goals. Few studies have identified knee OA phenotypes based on variables of activity limitation [18, 19]. Two studies used the Western Ontario and McMaster Universities Osteoarthritis Index (WOMAC) function subscale as the phenotypic variables [18, 19]. They reported that adults experiencing risk of knee OA and those with symptomatic knee OA demonstrated a variety of functional decline measured by the WOMAC function subscale. However, the phenotypes based on the sum score of 17 functional activities in WOMAC might not be accurately linked to the physical therapy management [20, 21]. The further analysis of the specific items of activities with greater difficulty would be needed.

Identifying specific activities impacted by knee OA should be more useful to guide management and prognosis. However, many activities are associated with knee OA symptoms. Stair climbing, rising from a chair and walking were reportedly the most common functional limitations among individuals with knee OA [22]. Moreover, the practice guidelines of knee OA suggest evaluating the functional capacity of walking, stair climbing, sit-to-stand, and balance ability [23]. Therefore, this study aimed to verify these activity limitations as reported in literature which could specifically classify the phenotypes of individuals with knee OA. The impairments and participation restrictions among phenotypes were also compared to validate the subgroup classification. We hypothesized that specific activity limitations would be identified and these activities could also classify individuals with symptomatic knee OA in homogeneous subgroups regarding level of disability. Moreover, the severity of impairments and participation restrictions would differ among phenotypes and these variables would be able to explain more variances when subgrouping of participants was considered.

## Methods

### Participants

**Fig. 1:**
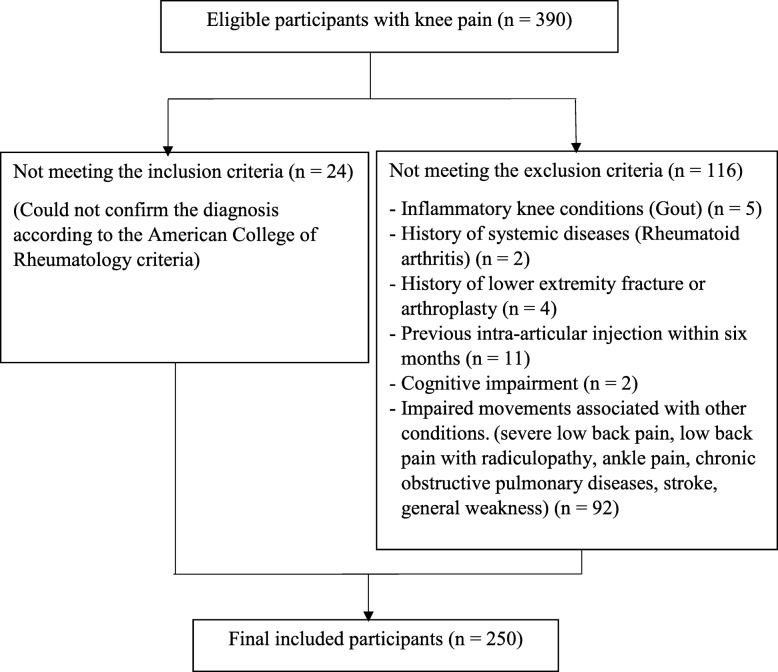
Participants recruitment process

The participants were enlisted from communities in the areas of services of ten physical therapy primary care settings in Thailand. They comprised individuals with symptomatic knee OA according to the American College of Rheumatology clinical criteria for knee OA [24]. The criteria included joint pain, and having three of the following criteria: 1) crepitus on active joint motion, 2) morning stiffness less than 30 min, 3) age more than 50 years, 4) bony enlargement of the knee, 5) bony tenderness of the knee and 6) no palpable warmth [25]. The exclusion criteria comprised participants having inflammatory knee conditions, history of systemic diseases, lower extremity fracture or arthroplasty, previous intra-articular injection within 6 months, cognitive impairment, or impaired movements associated with other conditions. Fig 1 presents the participants’ recruitment process. All participants provided written informed consent before collecting data.

### Knee OA assessments

An extensive assessment list for knee OA based on the ICF was used in this survey [26]. This list was developed using Delphi method consensus by ten physical therapy experts. Assessment tools comprised 16 categories of impairment (body function/body structure), and 33 categories of activity/participation limitation domains considered relevant to individuals with knee OA [26].

The impairment outcomes consisted of the worst pain during 48 h, muscle power of knee flexors and extensors, passive range of motion (PROM) of knee flexion and extension. The worst pain during 48 h was measured by numerical rating scale. Goniometry was used to measure PROM of knee flexion and extension in the supine lying position. Muscle power was determined according to standard manual muscle test on a 0 to 5 scale [27]. Hip, knee and ankle muscles were tested in supine, prone, sitting and standing positions.

Twenty-three activities from the comprehensive ICF core set for osteoarthritis were assessed including changing basic body position of lying down, changing and maintaining squatting, kneeling, sitting, standing, cross sitting, and side sitting positions, four walking patterns (short and long distance, around obstacles, and on different surfaces), stair climbing, toileting, taking off footwear, putting on and taking of pants, cleaning living area and remunerative employment. All these activities were subjectively examined by asking the participants to indicate the degree of difficulty of the activities during the past week. The rating of zero to four according to ICF qualifier guideline were used as 0 “no difficulty” (0–4%), 1 “mild difficulty” (5–24%), 2 “moderate difficulty” (25–49%), 3 “severe difficulty” (50–95%), and 4 “complete difficulty” (96–100%) [12]. The code of 9 as “not applicable” was also available. In addition, two common activity performance tests were also examined to quantify the activity abilities. The walking ability was assessed using timed 40-m self-paced walking and the stair climbing test which timing 5 steps ascending and descending the stair was also used [26].

Ten items of participation restriction, based on the ICF comprehensive core set for OA, were evaluated consisting of using private motorized transportation, using public motorized transportation, driving human-powered transportation, driving motorized vehicle, acquiring goods and services, assisting others in movement, community life, sports, hobbies, and socializing. The ICF qualifier procedure was also used to identify the level of difficulty to perform the participation items [12].

### Data analysis

Descriptive statistics were used to outline the personal characteristics of the participants including age, body mass index (BMI) and duration of knee pain. Three cluster variables were identified based on the results of the survey of 23 activities. The selected variables must have proper distributions of difficulty levels among 250 participants. These variables also had to be confirmed by the evidence from literature as the apparently important activity limitations among patients with knee OA.

Two-step cluster analysis was used to classify the phenotypes of knee OA due to the mixed types of cluster variables including both categorical and numeric variables. Optimal number of clusters, log-likelihood distance measure and Schwarz’s Bayesian Criterion were used [28].

To validate the phenotypes, the impairment and participation variables were compared. The Kruskal-Wallis test was used to compare pain intensity, knee flexion and extension PROM, muscle power of knee flexors and extensors, the level of difficulty of acquiring goods and services and community life among phenotypes. The statistical significance level was set at 0.05. The regression analysis was also performed to confirm the variance accounted for of impairment and participation variables for overall and subgroups of participants in each knee OA phenotype.

## Results

In all, 250 participants with symptomatic knee osteoarthritis (OA) were enrolled in this study. Age, BMI, duration of knee pain and proportion of males and females of overall participants and the subgroups from cluster analysis are presented in Table 1. The BMI were significantly different among subgroups.

**Table 1:** Demographic data of participants and the subgroups

	All participants (*N* = 250)	1 (No disability) (*N* = 79)	2 (mild disability) (*N* = 67)	3 (moderate disability) (*N* = 76)	4 (severe disability) (*N* = 28)	*P* - values
Age (years)	65.44 (8.70)	66.7 (7.97)	66.49 (9.85)	64.55 (8.35)	62.8 (6.76)	0.112
BMI (kg/m^2^)	26.07 (4.36)	25.13 (3.61)	25.30 (4.48)	27.37 (4.95)	27.07 (3.53)	0.003^a^
Duration of knee pain (years)	5.02 (3.78)	5.56 (4.24)	5.27 (3.72)	4.24 (3.14)	4.54 (3.18)	0.129
Gender (female/male)	216/34	67/12	57/10	68/8	24/4	.858

**Note:** Age, BMI and duration of pain were compared among subgroups using one-way ANOVA. Gender proportion was compared using Chi-square

^a^different among subgroups at *p* < .05. Post-Hoc analysis showed differences between group 1 & 3, group 2 & 3, and group 2 & 4

**Fig. 2:**
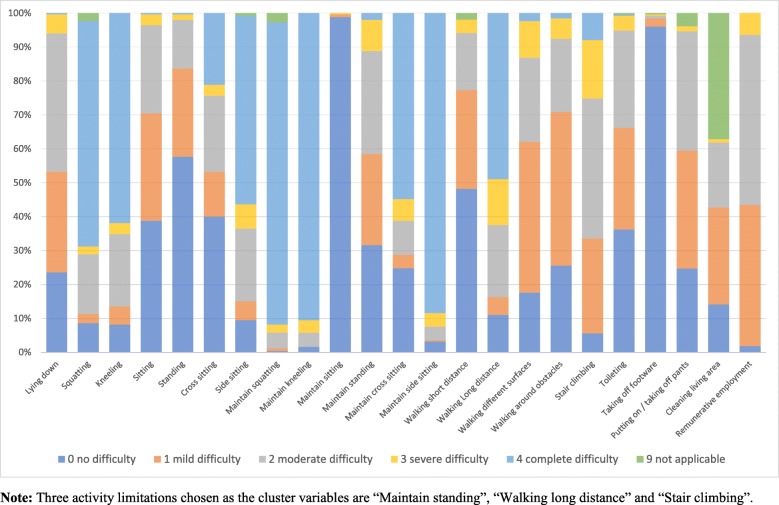
The distributions of the responses of 23 activity limitations assessed

Fig. 2 presents the distributions of the responses of 23 activities limitation assessed. Three activities chosen based on literature to possibly be the cluster variables including maintaining standing position, walking long distance and stair climbing met the criteria of 100% responses and had appropriate distributions of difficulties among 250 participants. Therefore, they were taken into the cluster analysis to further determine the phenotypes of individuals with knee OA. The results of performance tests of timed self-paced walking and stair climbing test were used for the analysis.

### Cluster variables and phenotypes of knee OA

The results of cluster analysis showed that three chosen variables were appropriate for grouping the individuals with knee OA into phenotypes. All 250 cases were entered in the analysis. As presented in Table 2, the results showed four phenotypes with good cluster quality (average silhouette = 0.5). The most important predictors for the cluster membership were maintaining a standing position (1.0) followed by stair climbing time (0.05). The least important was walking time (0.04). The results showed four phenotypes associated with the degree of activity limitation consisting of no (31.6%), mild (26.8%), moderate (30.4%), and severe disability (11.2%).

**Table 2:** Phenotypes according to the clustered activity limitations

	Cluster			
	1 (No disability) (***N*** = 79)	2 (mild disability) (***N*** = 67)	3 (moderate disability) (***N*** = 76)	4 (severe disability) (***N*** = 28)
the difficulty level of maintaining a standing position: median (Q1, Q3)	0 (0,0)	1(1,1)	2 (2,2)	3 (3,3)
stair climbing time (seconds): mean	12.43	15.29	18.01	18.23
walking time (seconds): mean	53.72	58.51	63.37	71.02

After the subgroups were identified, the impairment variables consisting of pain intensity, PROM of knee flexion and extension and strength of knee flexor and extensor muscles were compared among phenotypes. The results are presented in Table 3 as all variables significantly differed among homogeneous subgroups of knee OA (*p* <  0.05). Two out of 10 variables of participation restriction domain which rated by more than 80% of participants were also used for the verification by comparisons among phenotypes. Both acquiring goods and services and community life demonstrated a significant difference among phenotypes.

**Table 3:** The comparisons of impairment and participation restriction variables among phenotypes

	Cluster 1 (3) (*N* = 79)	Cluster 2 (4) (*N* = 67)	Cluster 3 (2) (*N* = 76)	Cluster 4 (1) (*N* = 28)	*p*-value^a^
Pain scale: Mean ± SD	3.88 ± 2.68	4.06 ± 2.44	4.82 ± 2.61	5.18 ± 3.20	0.012*
FPROM (degree): Mean ± SD	131.22 ± 9.92	127.69 ± 12.62	123.42 ± 16.93	118.96 ± 24.58	0.001*
EPROM (degree): Mean ± SD	3.19 ± 4.92	5.48 ± 5.73	5.25 ± 5.95	5.64 ± 6.18	0.032*
Knee flexor muscle power: Median (Q1, Q3)	4 (4,5)	4 (4,5)	4 (3.25,5)	4 (4,5)	0.003
Knee extensor muscle power: Median (Q1, Q3)	5 (4,5)	5 (4,5)	4 (4,5)	5 (4,5)	< 0.001
Acquisition of good and service: Median (Q1, Q3)	1 (0,2)	2 (1,2)	2 (2,3.75)	3 (2,4)	< 0.001
Community life: Median (Q1, Q3)	1 (0,2)	2 (1,2)	2 (2,3)	3 (1,4)	< 0.001

^a^*p*-values of the Kruskal-Wallis test

Abbreviation: *FPROM* Flexion passive range of motion, *EPROM* Extension passive range of motion. *different among clusters at p < .05

Table 4 presents the variance accounted-for statistic models of impairment and participation variables for the activity limitations used as cluster variables among all participants and each cluster. There were marked that the subgroups had greater variance accounted for all three activity limitations compared with the overall participants.

**Table 4:** The variance accounted for statistical models of impairment and participation variables for the activty limitation used as cluster variables

Variables	Impairment and participation variables included in the model	R	Adjusted R^2^	F (*p* -value)
Maintaining a standing position				
All subject (*N* = 250)	Extensor, EPROM, Acquisition of good and service, Pain scale, FPROM, Flexor, Community life	.365	.108	5.317 (<.001)
Cluster 1 (*N* = 79)	Extensor, Pain scale, Acquisition of good and service, FPROM, EPROM, Flexor, Community life	.602	.139	1.624 (.186)
Timed walking				
All subject (*N*= 250)	Extensor, EPROM, Acquisition of good and service, Pain scale, FPROM, Flexor, Community life	.540	.272	14.258 (<.001)
Cluster 1 (*N* = 79)	Extensor, Pain scale, Acquisition of good and service, FPROM, EPROM, Flexor, Community life	.713	.337	2.957 (.027)
Cluster 2 (*N* = 67)	Extensor, EPROM, Acquisition of good and service, Pain scale, FPROM, Community life, Flexor	.619	.319	6.021 (<.001)
Cluster 3 (*N* = 76)	Extensor, Acquisition of good and service, Pain scale, Flexor, FPROM, EPROM, Community life	.470	.144	2.874 (.011)
Cluster 4 (*N* = 28)	Extensor, FPROM, Pain scale, EPROM, Community life, Flexor, Acquisition of good and service	.550	.220	3.665 (.002)
Timed stair test				
All subject (*N* = 250)	Extensor, EROM, Acquisition of good and service, Pain scale, FPROM, Flexor, Community life	.563	.297	16.057 (<.001)
Cluster 1 (*N* = 79)	Extensor, Pain scale, Acquisition of good and service, FPROM, EPROM, Flexor, Community life	.744	.397	3.540 (.012)
Cluster 2 (*N* = 67)	Extensor, EPROM, Acquisition of good and service, Pain scale, FPROM, Community life, Flexor	.597	.290	5.366 (<.001)
Cluster 3 (*N* = 76)	Extensor, Acquisition of good and service, Pain scale, Flexor, FPROM, EPROM, Community life	.433	.108	2.345 (.033)
Cluster 4 (*N* = 28)	Extensor, FPROM, Pain scale, EPROM, Community life, Flexor, Acquisition of good and service	.495	.155	2.730 (.016)

**Note:** The model for cluster 2, 3 and 4 of the “Maintaining a standing position” could not be computed since the dependent variables are constant

**Abbreviation:***FPROM* Flexion passive range of motion, *EPROM* Extension passive range of motion, *Extensor* Knee extensor muscle power, *Flexor* Knee flexor muscle power

## Discussion

This study aimed to identify the activity limitations appropriate to identify homogeneous subgroups of knee OA. The impairment and participation restriction were then compared among the established subgroups. The results were congregant with our hypotheses. Three variables including difficulty levels of maintaining a standing position, walking time, and stair climbing time represented distinct phenotypes. Four phenotypes were identified with differing levels of disability in knee OA. The impairments and participation restriction levels significantly differed among individuals in each phenotype. Greater variance was accounted for when using the subgroups according to the phenotype compared with the overall group. This implied that the subgroup could more precisely identify people not only activity but also their impairments and participation levels. The related literature also confirmed that among patients with knee OA, these three activities were the common ones being assessed and used as functional goals in physical therapy clinics [13, 22, 29]. Therefore, the three activity limitations identified in this study had the power to classify subgroups of people with knee OA.

The activity limitation variables had been used to classify individuals with knee OA in homogeneous subgroups [18, 19]. However, the number of phenotypes varied among studies. In this study, activity limitation variables could cluster the patients with knee OA in four phenotypes with the disability levels of no, mild, moderate and severe. A related study conducting 5-year follow-up among people with early symptomatic knee OA identified three phenotypes of knee OA consisting of good, moderate, and poor outcome subgroups [18]. The different characteristics regarding activity limitation decline over time among subgroups was reported. The authors suggested that their homogenous identification of individuals with knee OA could be used to develop specific interventions [18]. Another 7-year follow-up study proposed five phenotypes of people with knee OA differentiated by functional decline. The subgroups comprised high functioning, minimal limitation, late worsening, remitting and progressive worsening trajectory [19]. Secondary analysis demonstrated an association between decline of activity and contributing factors of activity limitation including radiographic disease severity, knee pain, obesity and depressive symptom [19].

Other studies have proposed using commonly used functional scores such as WOMAC to identify subgroups of knee OA [6, 30]. However, the WOMAC function subscale could not appropriately predict phenotypes [6, 30]. The study by Egsgaard et al used mixed phenotypic variables including WOMAC subscales, Lequesne index, quality of life, pain catastrophizing, quantitative sensory testing and inflammatory profiles as the clustering variables. Only the pain sensitization profile could identify four distinct phenotypes comprising low sensitivity to pain, early phase sensitization, presence of pain sensitization and presence of pain sensitization and catastrophizing [30]. On the other hand, the secondary comparison of activity limitations among the phenotypes of impairment variables also significantly differed [31, 32]. This implied a close relationship among the activity limitations and impairments among individuals with knee OA.

In this study, the results of secondary analysis supported that activity limitation variables identified in the cluster analysis could classify participants with knee OA in homogeneous subgroups. The comparisons of impairment and participation restriction variables demonstrated significant differences among phenotypes of all variables. Pain intensity, limited ROM, muscle power and participation restriction levels were worse in subgroups with greater disability accordingly. The impairments related to the activity limitations of each phenotype might be used to develop specific treatment guidelines. Similarly, the related study demonstrated that knee pain and knee flexion ROM also differed among phenotypes based on activity limitations [18].

The major strength of this study was that it identified the common activities which would be disable in persons with knee OA. These activities would be useful in the goal setting process of rehabilitation in patients with different levels of disabilities. However, the study had some limitations. First, the participants included were the patients registered to the primary healthcare settings and diagnosed using the knee OA clinical criteria. No radiographic or other investigations were performed to confirm the structural lesions. Second, due to the cross-sectional nature, the cause and effect of the impairments and participation variables on activity limitations could not be identified.

## Conclusion

The cluster analysis confirmed the heterogeneity nature of knee OA. The three activity limitation variables of maintaining a standing position, stair climbing time and walking time could be used to identify homogeneous subgroups of knee OA. Goal setting and treatment planning could be guided by the characteristics of phenotype. However, specific physical therapy management guidelines related to phenotypes are required for further study to identify those completely related factors of these activity limitations.

## Data Availability

The datasets used and analyzed during the current study are available from the corresponding author upon reasonable request.

## Abbreviations

OA: Osteoarthritis
ICF: International classification of functioning, disability and health
ROM: Range of motion
WOMAC: The Western Ontario and Mcmaster Universities osteoarthritis index
PROM: Passive range of motion
BMI: Body mass index
FPROM: Flexion passive range of motion
EPROM: Extension passive range of motion
